# Identification and Evolution of TGF-β Signaling Pathway Members in Twenty-Four Animal Species and Expression in Tilapia

**DOI:** 10.3390/ijms19041154

**Published:** 2018-04-11

**Authors:** Shuqing Zheng, Juan Long, Zhilong Liu, Wenjing Tao, Deshou Wang

**Affiliations:** Key Laboratory of Freshwater Fish Reproduction and Development (Ministry of Education), Key Laboratory of Aquatic Science of Chongqing, School of Life Sciences, Southwest University, Chongqing 400715, China; zhengsq0825@163.com (S.Z.); 18883398791@163.com (J.L.); bioliu545@163.com (Z.L.); enderwin@163.com (W.T.)

**Keywords:** transforming growth factor β signaling pathway, evolution, teleosts, tissue distribution, gonadal expression profile

## Abstract

Transforming growth factor β (TGF-β) signaling controls diverse cellular processes during embryogenesis as well as in mature tissues of multicellular animals. Here we carried out a comprehensive analysis of TGF-β pathway members in 24 representative animal species. The appearance of the TGF-β pathway was intrinsically linked to the emergence of metazoan. The total number of TGF-β ligands, receptors, and smads changed slightly in all invertebrates and jawless vertebrates analyzed. In contrast, expansion of the pathway members, especially ligands, was observed in jawed vertebrates most likely due to the second round of whole genome duplication (2R) and additional rounds in teleosts. Duplications of *TGFB2*, *TGFBR2*, *ACVR1*, *SMAD4* and *SMAD6*, which were resulted from 2R, were first isolated. Type II receptors may be originated from the *ACVR2*-like ancestor. Interestingly, *AMHR2* was not identified in Chimaeriformes and Cypriniformes even though they had the ligand *AMH*. Based on transcriptome data, TGF-β ligands exhibited a tissue-specific expression especially in the heart and gonads. However, most receptors and smads were expressed in multiple tissues indicating they were shared by different ligands. Spatial and temporal expression profiles of 8 genes in gonads of different developmental stages provided a fundamental clue for understanding their important roles in sex determination and reproduction. Taken together, our findings provided a global insight into the phylogeny and expression patterns of the TGF-β pathway genes, and hence contribute to the greater understanding of their biological roles in the organism especially in teleosts.

## 1. Introduction

**Figure 1:**
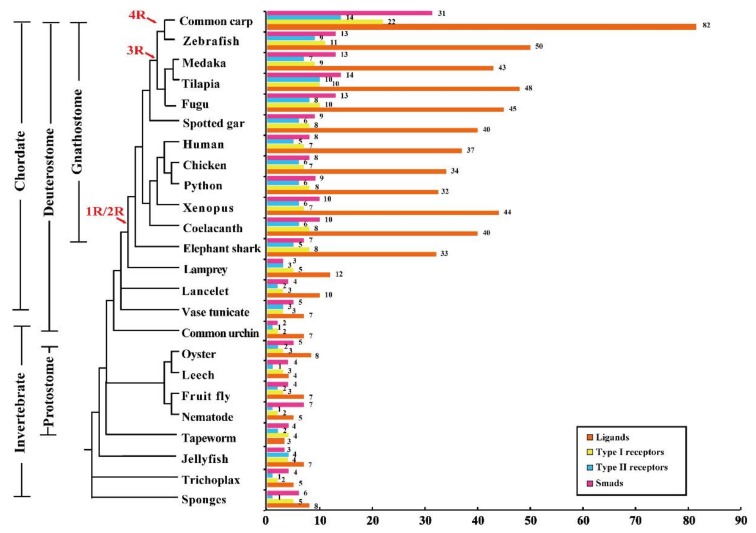
Phylogenetic relationship of 24 representative animals analyzed and numbers of transforming growth factor β (TGF-β) pathway gene. 1R, 2R, 3R and 4R indicate the four rounds of WGD that occurred during vertebrate evolution. The complete TGF-β pathway was first appeared in sponges, and the pathway members are universally present in all metazoans.

The evolution of animals was accompanied by an increase in systematic complexity including highly specialized tissues and organs. Gene duplication has been suggested to be a primary mechanism for the increase of organismal complexity and the generation of evolutionary novelty [1]. Whole genome duplication (WGD) is one of the important mechanisms which can rapidly generate duplicate copies of genes in species evolution [2]. Many families of genes such as nuclear receptors (NRs), Wnt-protein ligands and GPCRs are known to have evolved through WGDs [3,4]. Despite being controversial, it is widely accepted that three rounds of WGD occurred during vertebrate evolution. The first two rounds of duplication events (1R and 2R) occurred early in the vertebrate lineage, while the third event (3R) only occurred in teleosts [5,6,7,8]. Some teleosts, such as rainbow trout and common carp [9,10], even have undergone the fourth round of genome duplication (4R) (Figure 1). After duplication, divergence both in expression patterns and protein sequences (namely subfunctionalization and neofunctionalization) can be responsible for the retention of gene duplicates [11,12]. However, massive gene loss typically followed WGDs, and in most cases only a single copy of the duplicated genes will be retained [13]. Thus, it is hard to interpret molecular phylogenies due to gene loss among distantly related lineages.

The transforming growth factor β (TGF-β) signaling pathway consists of extracellular ligands (including TGF-β-like group and BMP-like group), cell surface receptors (including type I and type II serine-threonine kinase receptors), and intracellular smad proteins (including receptor-regulated smad (R-SMAD), common-smad (Co-SMAD), and inhibitory smad (I-SMAD)) [14,15]. TGF-β ligands comprise a secretion signal peptide, a ~250-residue prodomain, and a ~110-residue growth factor domain which encompasses 6–9, and usually 7, conserved cysteines. 6 of the characteristic cysteine residues form 3 intramolecular disulfide bonds, and the seventh cysteine forms a intermolecular disulfide bond linking two monomers into a dimer [16,17]. This dimer binds to specific tetrameric type II/type I kinase receptor complexes, which stabilizes and activates type I receptors. The activated type I receptors then transduce the signals by phosphorylating the R-SMADs. In general, TGF-β-like group generally phosphorylate SMAD2 and SMAD3, whereas BMP-like group generally induce phosphorylation of SMAD1, SMAD5 and SAMD8 [18,19]. The activated R-SMADs form hetero-oligomeric complexes with Co-SMAD (SMAD4), which are translocated to the nucleus where they regulate the expression of target genes. I-SMADs (SMAD6 and SMAD7) can inhibit R-SMAD activation by type I receptors.

The TGF-β pathway members are conserved in organisms ranging from Nematoda and Arthropoda to Mammalia [20]. As for ligands, there are 9 genes in *Mnemiopsis leidyi* [21], 7 in *Drosophila melanogaster* [22], 5 in *Caenorhabditis elegans* [23], and at least 30 in mammals [17,24]. Two functional classes of receptors, type I and type II, which are encoded by 7 (*ALK1*-*ACVRL1*, *ALK2*-*ACVR1*, *ALK3*-*BMPR1A*, *ALK4*-*ACVR1B*, *ALK5*-*TGFBR1*, *ALK6*-*BMPR1B*, *ALK7*-*ACVR1C*) and 5 (*BMPR2*, *ACVR2A*, *ACVR2B*, *AMHR2*, and *TGFBR2*) genes in mammals, respectively [25,26]. *Drosophila* species contain three type I and two type II receptors. Extensive research on the repertoires of smads has been reported in zebrafish (12), medaka (12), fugu (13) and the green spotted puffer (14) [27]. However, to date, the origin and evolution of whole TGF-β pathway members in the animal kingdom are still unclear because of scant analysis of this pathway in representative animal species due to lack of genome data. Recently, genomes of representative species have been sequenced and opened, such as sea lamprey (a jawless cyclostomata) [28], elephant shark (a chondrichthyan) [29], coelacanth (an early sarcopterygian) [30], spotted gar (a non-teleost actinopterygian) [31] and many teleosts. The completion of genome sequencing of these species provides new resources for tracing the evolution of this pathway.

TGF-β pathway members create the networks that establish multicellular animal body plans. They specify anteroposterior, dorsoventral (bilateral), left–right axes, details of individual organs and regulate development and homeostasis [32,33,34,35,36]. The influence of the TGF-β pathway members on fertility and reproduction in organisms as diverse as flies and humans is impressive [37]. Additionally, several recent studies have suggested that TGF-β pathway members were involved in sex determination in fishes. These included *gsdf* [38,39,40,41], *bmp15* [42], *amhr2* [43] and Y-linked duplicates of the *amh* (*amhy*) [44,45], *gsdf* (*gsdf^Y^*) [46] and *gdf6* (*gdf6^Y^*) [47].

However, to date, analyses of this pathway have so far been restricted to ligands, receptors or smads alone in one or several species. Expression data are available only for single or a subfamily of the TGF-β in a particular tissue or at a single stage of development [48,49]. The expression profiles of most of the TGF-β pathway genes in multiple tissues are still lacking. Moreover, other than studies in human and model organisms, little is known about the evolution and expression of TGF-β pathway members in other species, especially in teleosts. To address these issues, we isolated TGF-β pathway members from 24 representative animal species, performed phylogenetic analyses of the ligands, receptors and smads, and analyzed the spatial-temporal gene expression profiles in different tissues especially gonads of a teleost, the Nile tilapia.

## 2. Results

### 2.1. TGF-β Pathway Genes across Different Animals

Comparative analyses of the TGF-β pathway genes in 24 representative animal species revealed that the complete functional TGF-β pathway, including multiple ligands, receptors and smads, appeared in the sponge *Amphimedon queenslandica*, an early diverging metazoan (Figure 1 and Table S1). In invertebrates and jawless vertebrates, the total number of TGF-β ligands, receptors and smads changed slightly. Jawed vertebrates (gnathostomes) had an expanded set of ligands, receptors and smads most likely due to 2R event. Duplications of TGF-β members were also observed from 3R in teleosts and 4R in common carp although many TGF-β pathway members were lost after duplication. Additionally, the number of TGF-β ligands greatly exceeded the number of two type receptors and smads in jawed vertebrates. The accession numbers of all TGF-β pathway members we analyzed are listed in Table S2. Genomic distribution of TGF-β pathway members in tilapia was shown in Figure S1.

**Figure 2:**
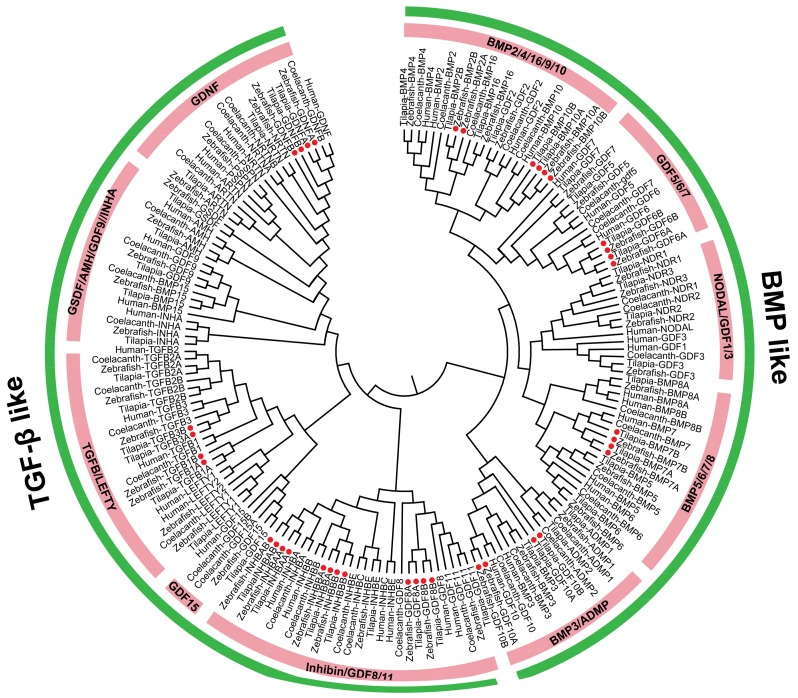
Phylogenetic tree of TGF-β ligands from tilapia, zebrafish, coelacanth, and human. The TGF-β ligands were clustered into TGF-β-like and BMP-like clades, with each clade can be subdivided into many different small groups. The ML method was used to construct the tree by MEGA 6.0 software. The amino acid sequences of TGF-β peptide domains were aligned using the multiple alignment software Bioedit. Red dots indicate genes which retained two copies in teleosts after 3R events. GenBank accession numbers of the sequences used are listed in Table S2a.

TGF-β ligands were clustered into TGF-β-like and BMP-like clades from phylogenetic tree we generated based on mature TGF-β peptide domains. Each clade can be subdivided into many different small groups (Figure 2). There are about 43–50 ligands in teleosts, of which 11 genes (*TGFB1*, *TGFB3*, *BMP2*, *BMP7*, *BMP10*, *GDF6*, *GDF8*, *GDF10*, *INHBA*, *INHBB* and *GDNF*) marked with red dots retained two copies in teleosts which were originated from 3R. Additionally, there were 5 genes (*NDR1*, *BMP8*, *ADMP*, *TGFB2* and *NRTN*) which had two copies in some 2R species. For instance, duplication of *TGFB2* was found for the first time in the elephant shark, coelacanth, and python (Figure S2a). Additionally, *BMP16*, a relative of *BMP2/4*, was first identified in whale shark in this study (Figure S2b).

**Figure 3:**
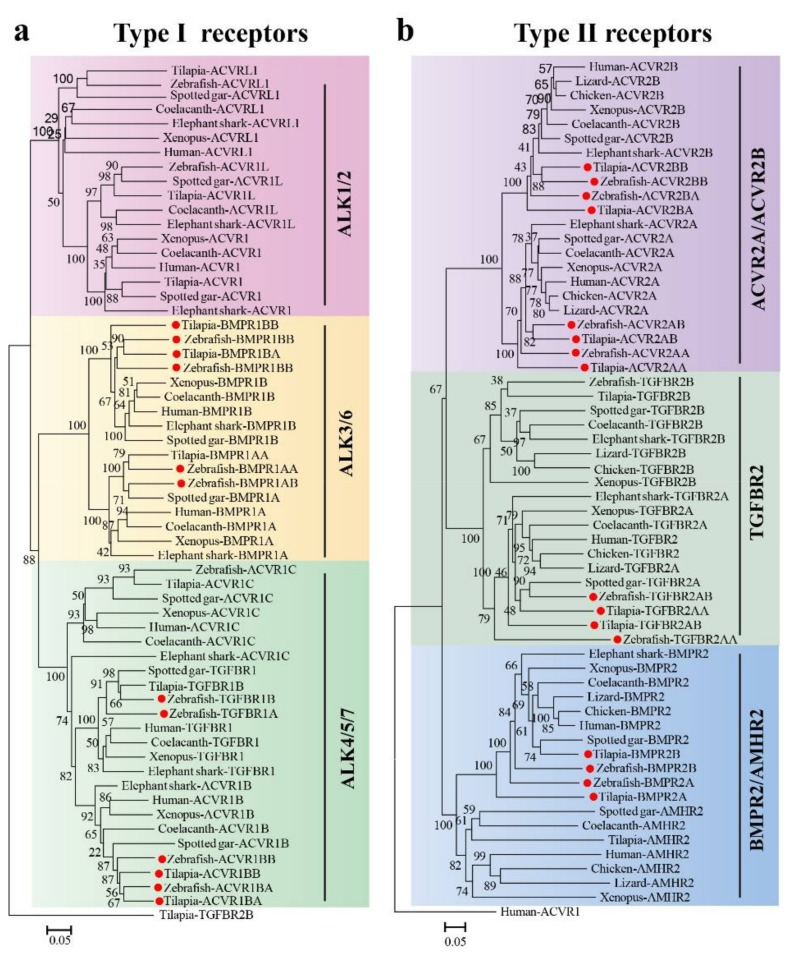
Phylogenetic trees of TGF-β type I (**a**) and type II (**b**) receptors. The trees were constructed using the same method as described in Figure 2. (**a**) Type I receptors from elephant shark, coelacanth, spotted gar, xenopus, human, zebrafish and tilapia were clustered into ALK1/2, ALK3/6 and ALK4/5/7 three branches, tilapia TGFBR2B was used as an outgroup; (**b**) Type II receptors from elephant shark, coelacanth, spotted gar, xenopus, lizard, chicken, human, zebrafish and tilapia were clustered into ACVR2A/ACVR2B, TGFBR2 and BMPR2/AMHR2 three branches, human ACVR1 was used as an outgroup. Red dots indicate genes which retained two copies in teleosts after 3R events. GenBank accession numbers of the sequences used are listed in Table S2b,c.

TGF-β type I receptors can be divided into ALK1/2, ALK3/6 and ALK4/5/7 subfamilies (Figure 3a). In teleosts, we isolated 9–11 potential type I receptors. *ALK3/4/5/6* had two copies in teleosts, while only one in tetrapods. Moreover, *ACVR1L*, a copy gene of *ALK2*, was isolated in the shark, coelacanth, and spotted gar in this study. Type II receptors can be divided into TGFBR2, ACVR2A/ACVR2B and BMPR2/AMHR2 subfamilies (Figure 3b). In teleosts, we identified 7–10 type II receptors. All orthologous genes of mammals have two copies in teleosts except *AMHR2*. In addition, *AMHR2* was not found in Chimaeriformes (elephant shark, whale shark) and Cypriniformes, at least in Cyprinidae (zebrafish, common carp, grass carp and bluntnose black bream) genomes which had the corresponding ligand *AMH* (Figure S3). In addition, duplication of *TGFBR2* (*TGFBR2B*) was found not only in teleosts but also in birds, amphibians, reptiles, cartilage, and bony fishes.

**Figure 4:**
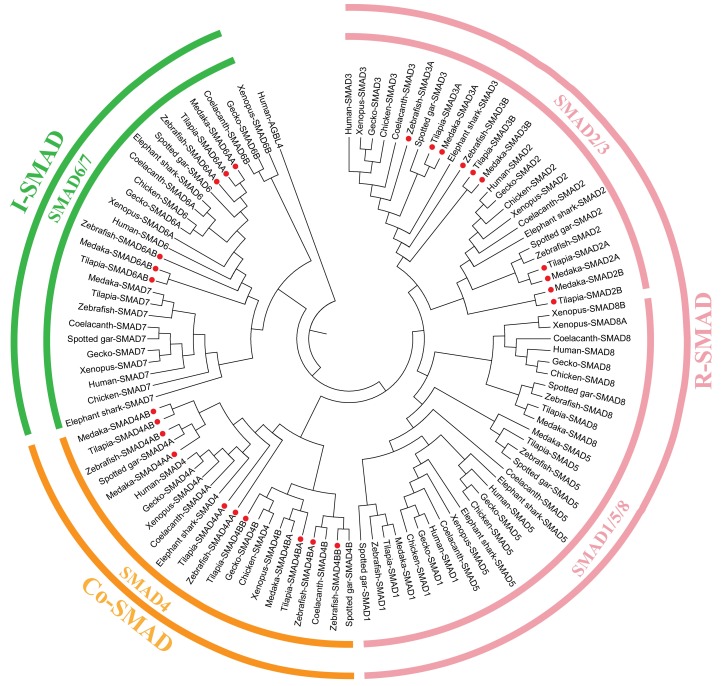
Phylogenetic tree of smads. The tree was constructed using the same method as described in Figure 2. Smads from elephant shark, coelacanth, spotted gar, xenopus, gecko, chicken, human, zebrafish, and tilapia were clustered into R-SMAD (SMAD1/5/8, SMAD2/3), Co-SMAD (SMAD4) and I-SMAD (SMAD6/7) branches, human AGBL4 was used as an outgroup. Red dots indicate genes which retained two copies in teleosts after 3R events. GenBank accession numbers of the sequences used are listed in Table S2d.

**Figure 5:**
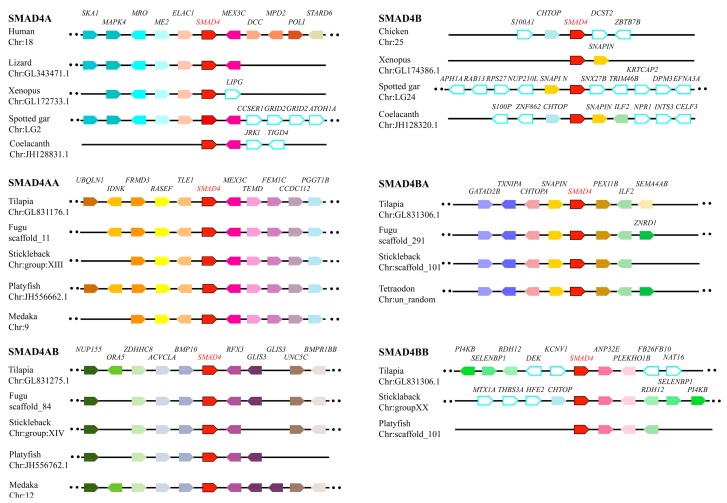
Syntenic analysis of *SMAD4* genomic region. Genomic regions flanking SMAD4 copies were analyzed in representative vertebrate species including tetrapods (human, chicken, lizard, and xenopus), sarcopterygians (coelacanth) and actinopterygians (spotted gar, tilapia, fugu, stickleback, medaka, platyfish, and tetraodon) using Genomicus browser. The chromosome or scaffold number was indicated under the species name. The bar lengths were not proportional to the distances between genes. Dotted lines indicated omitted genes in the chromosome or scaffold. Different genes were represented by different colored pentagons and SMAD4 copies were indicated in red. The direction of pentagons indicated the gene direction. The gene names were placed on top of the pentagons.

In teleosts, smads were clustered into 3 subfamilies with 6–7 R-SMADs, 3–4 Co-SMADs and 3 I-SMADs (Figure 4). Duplications of *SMAD2*/*3* derived from 3R event, while *SMAD1*/*5*/*7*/*8* had only one copy in all species analyzed except xenopus in which two *SMAD8* were identified. Phylogeny analysis of *SMAD4* indicated that it has undergone 2R event to form 2 copies in coelacanth, spotted gar and xenopus, and have 4 in teleosts derived from 3R (Figure S2c). To further elucidate the *SMAD4* evolutionary history, we characterized and compared the adjacent genomic regions of each *SMAD4* paralogous gene loci in representative vertebrate species including tetrapods (human, chicken, lizard, and xenopus), sarcopterygians (coelacanth) and actinopterygians (spotted gar, tilapia, zebrafish, medaka, fugu, stickleback, platyfish and tetraodon) (Figure 5). Synteny analysis indicated that two *SMAD4*, namely *SMAD4A* and *SMAD4B*, were conserved in coelacanth, spotted gar and xenopus. Human and lizard retained *SMAD4A*, while chicken retained *SMAD4B*. The genomic regions of *SMAD4A* and *SMAD4B* have been duplicated in teleosts, likely due to the teleost 3R. Conserved synteny of 3R-duplicated genes in this region include *MEX3C* and *SNAPIN*. Furthermore, duplication of *SMAD6* (*SMAD6B*) was found in coelacanth, xenopus, python, and gecko. *SMAD6B* in these species were clustered together but branched from *SMAD6A* of the vertebrates (Figure S2d).

### 2.2. Tissue Distribution and Temporal Expression of TGF-β Pathway Members in the Tilapia Gonads

**Figure 6:**
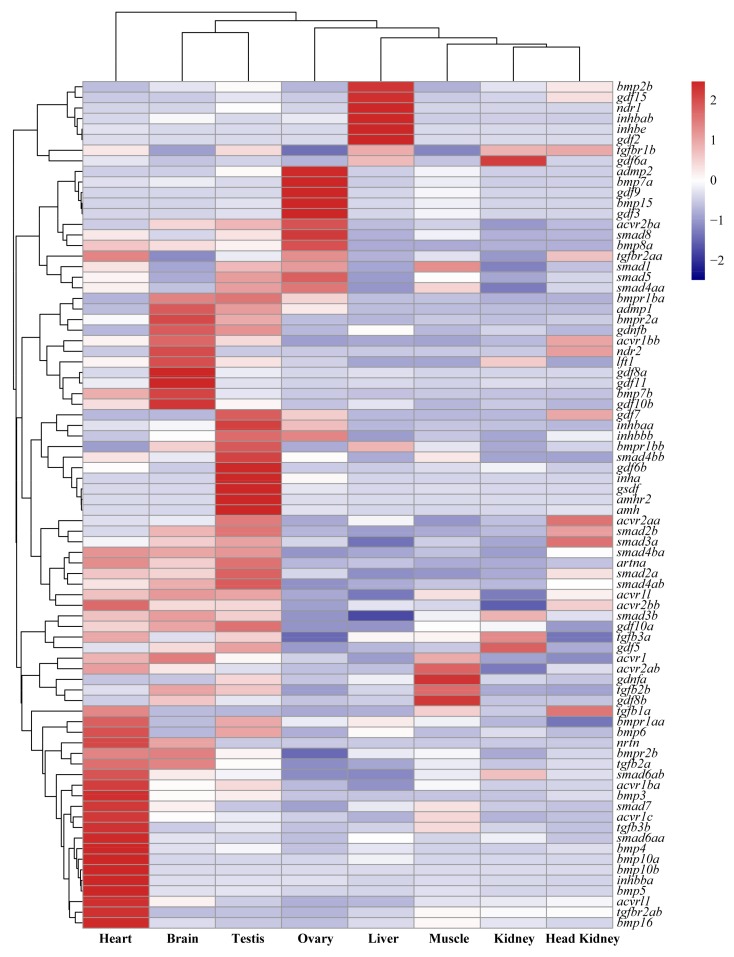
Tissue distribution (FPKM: fragments per kilobase of exon per million fragments mapped) of TGF-β pathway members in eight tissues of tilapia based on transcriptome data. A heat map showing expression of TGF-β pathway members in tilapia eight tissues, with red and blue indicating high and low expression, respectively. Each row represents a different gene, and each column represents an independent tissue sample. Hierarchical clustering of both genes and tissues (by correlation) organized the data set into similar expression patterns. The widespread complex expression patterns in all tissues were readily discernable. Most receptors and smads were expressed in multiple tissues while the ligands exhibited tissue-specific expression pattern especially in the heart and gonads.

A hierarchical cluster analysis based on transcriptome data of 8 adult tissues indicated that of the 80 identified TGF-β superfamily members in tilapia, 25 were expressed at background level in all 8 tissues according to the threshold we set. Most of smads and two types of receptors were expressed in multiple tissues, while TGF-β ligands exhibited a tissue-specific expression pattern with 8 ligands in the heart, 5 in the ovary, 4 in the testis and 3 in the liver (Figure 6).

**Figure 7:**
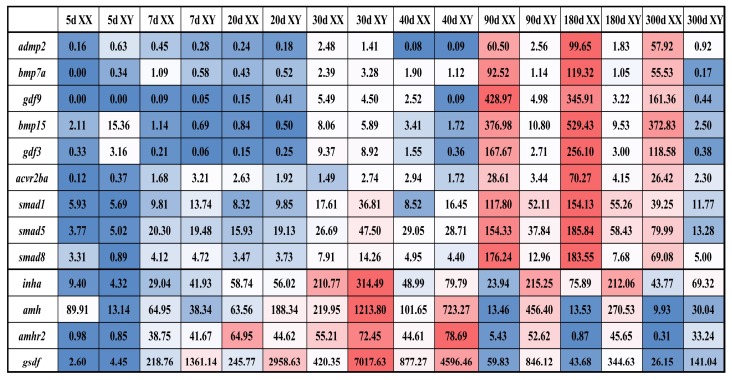
The expression profiles (FPKM) of TGF-β pathway members in the tilapia gonads. *admp2*, *bmp7a*, *gdf9*, *bmp15*, *gdf3*, *acvr2ba*, *smad1*, *smad5* and *smad8* were ovary-enriched genes, while *inha*, *gsdf*, *amh* and *amhr2* were testis-enriched genes. Red and blue indicate high and low expression, respectively.

Based on transcriptome data, 13 genes highly expressed in gonads were selected for analysis of their temporal expression patterns at different developmental stages (Figure 7). The expression levels of 9 ovary-enriched genes (*admp2*, *bmp7a*, *gdf9*, *bmp15*, *gdf3*, *acvr2ba*, *smad1*, *smad5*, *smad8*) were found to peak at 180 dah (days after hatching), followed by 90 and 300 dah, always higher in XX than XY gonads, while at relatively low level at early stages of gonad development (5, 7, 20, 30 and 40 dah) with little difference between XX and XY gonads. Expression of 4 testis-enriched genes (*inha*, *gsdf*, *amh* and *amhr2*) was gradually elevated from 5 dah, significantly elevated from 20 dah and peaked at 30 dah. At the later stages, their expression levels were gradually decreased.

### 2.3. Validation of Expression Profile of TGF-β Pathway Members by qPCR and Cellular Location by In Situ Hybridization (ISH) and Immunohistochemistry (IHC)

**Figure 8:**
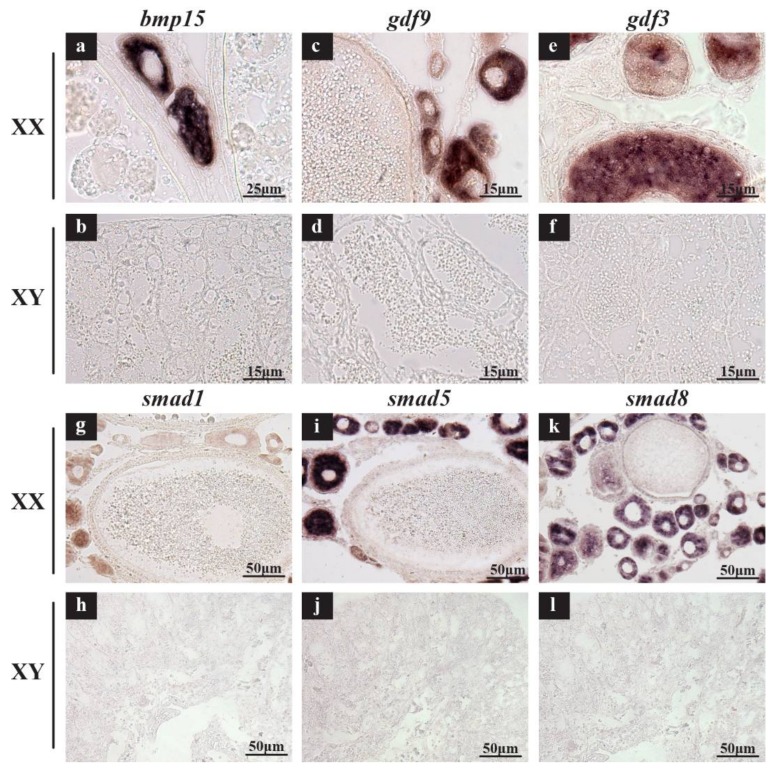
Cellular location of 6 TGF-β pathway genes in tilapia gonads at 180 dah by ISH. Specific signals (brown) of *bmp15*, *gdf9*, *gdf3*, *smad1*, *smad5* and *smad8* were observed in the cytoplasm of oocytes in the ovary (**a**,**c**,**e**,**g**,**i**,**k**). In contrast, no signal of these genes was observed in any cells of the testis (**b**,**d**,**f**,**h**,**j**,**l**). Scale bar: (**a**) 25 µm; (**b**–**f**) 15 µm; (**g**–**l**) 50 µm.

**Figure 9:**
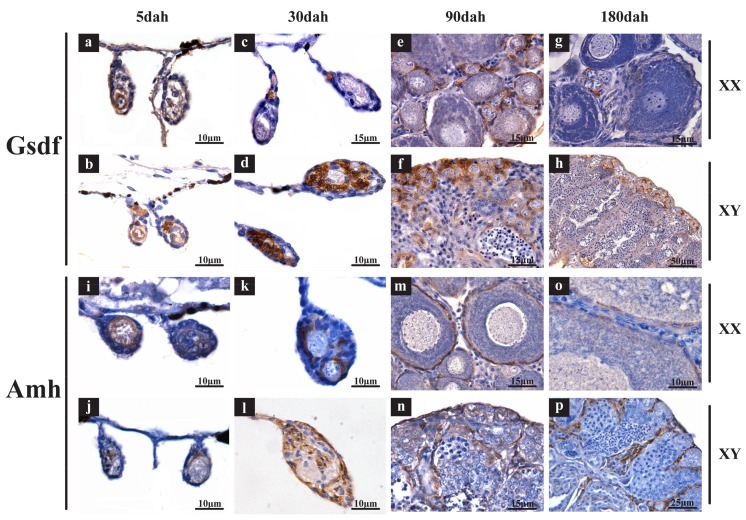
Cellular location of Gsdf and Amh in gonads by IHC. Gsdf-specific immunostaining was clearly observed in the somatic cells neighboring oogonia of XX gonads and Sertoli cells neighboring spermatogonia of the XY gonads at all stages (**a**–**h**). The Amh proteins were located in granule cells in ovary at all stages and somatic cells surrounding germ cells in the testis at 5 dah, and at later stages in myoid cells and Sertoli cells (**i**–**p**). Scale bar: (**a**,**b**,**d**,**i**–**l**,**o**) 10 µm; (**c**,**e**–**g**,**m**,**n**) 15 µm; (**p**) 25 µm; (**h**) 50 µm.

To verify the accuracy of transcriptome data, two genes were selected for qPCR validation of their expression. Additionally, ISH and IHC were performed for several highly expressed genes to detect their cellular location in gonads. Consistently, by qPCR, the expression levels of *bmp15* and *gdf9* were gradually elevated during gonadal development with much higher expression in XX than XY gonads at all stages (Figure S4). By ISH, strong signals (brown) of *bmp15*, *gdf9*, *gdf3*, *smad1*, *smad5* and *smad8* were observed in the cytoplasm of oocytes in the ovary (Figure 8a,c,e,g,i,k), while no signal of these genes was observed in any cells of the testis (Figure 8b,d,f,h,j,l). Slides did not show a positive signal in the negative controls of all genes hybridized with sense probes (Figure S5). By IHC, Gsdf-specific immunostaining was clearly observed in the somatic cells neighboring oogonia of the XX gonads and Sertoli cells neighboring spermatogonia of the XY gonads at 5, 30, 90 and 180 dah (Figure 9a–h). The Amh proteins were located in granulosa cells in the ovary at all stages and somatic cells surrounding germ cells in the testis at 5 dah, and later stages in myoid cells and Sertoli cells (Figure 9i–p). Negative controls with the primary antibody replaced with normal rabbit serum were shown in Figure S6.

## 3. Discussion

The growing number of sequenced species genomes provides more resources for understanding the evolution of gene families and developmental signaling pathways. Here, we focused on the TGF-β pathway members, which play important roles in developmental specification, to retrace their origin and evolution, in particular gene duplications and losses. We also examined the expressions of all TGF-β pathway members in the eight different tissues and their ontogenic expression in XX/XY gonads of tilapia.

### 3.1. Evolution of the TGF-β Signaling Pathway

**Figure 10:**
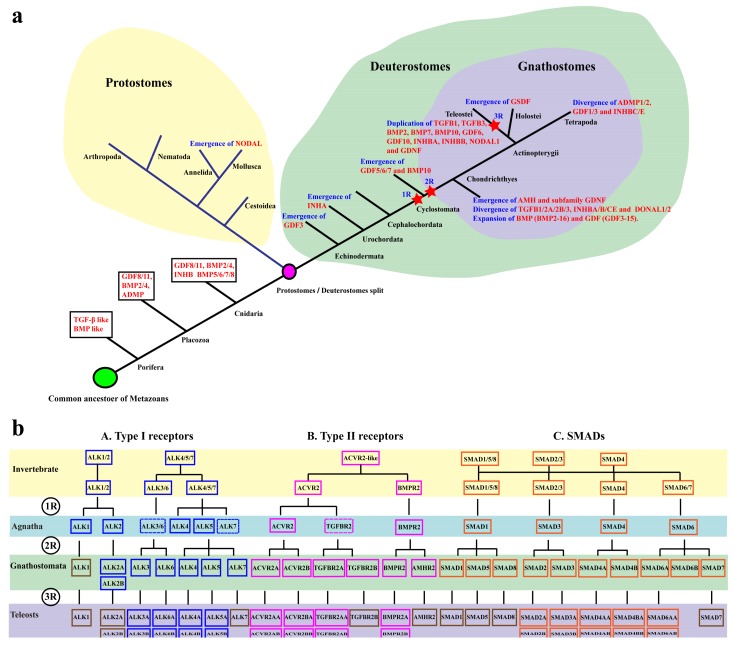
A schematic presentation of the evolution of TGF-β pathway members. 1R, 2R and 3R indicate the three rounds of WGD that occurred during vertebrate evolution. (**a**) Evolution of TGF-β ligands. The evolutionary tree is constructed with references from the tree of life web project (Available online: http://tolweb.org/tree/phylogeny.html). Branch lengths are not drawn to represent actual evolutionary distances. Protostomes, deuterostomes and gnathostomes were circled by yellow, green, and purple, respectively. Genes in the box indicate their presence in corresponding species. After split of protostome and deuterostome, changes (such as emergence, divergence, and duplication) of TGF-β ligands were mapped onto corresponding branches. The early emergence of these genes including, NODAL in Mollusca and Annelida, INHA in Urochordata, GDF5/6/7 in Cyclostomata were obtained from previous studies [62,63,64]. (**b**) Evolution of TGF-β type I receptors (A), type II receptors (B) and SMADs (C). The brown boxes indicate genes that with only one copy preserved after 2R or 3R. The dashed boxes indicate genes remain to be identified. The origins of the type I receptor can be traced to ALK1/2 and ALK4/5/7 in the vertebrate ancestor. Only one type II receptor *ACVR2* was found in invertebrate. *BMPR2* may be the paralog of *ACVR2* and the hypothetical origin of TGF-β type II receptors was *ACVR2*. The origin of the SMADs can be traced to SMAD1/5/8, SMAD2/3 and SMAD4 in early invertebrates. SMAD6/7 appeared later. Four SMADs, i.e., *SMAD1*, *SMAD3*, *SMAD4* and *SMAD6* appeared in agnatha. The whole SMAD family (SMAD1–8) were generated after 2R.

As has been proposed in the previous literature [21], our results supported that the appearance of the TGF-β pathway was intrinsically linked to the emergence of metazoan. In this study, we examined TGF-β pathway members in the genomes of 9 species of invertebrates and 15 species of chordates. We found that the diversity and total number of TGF-β ligands, receptors and smads only varied slightly in all invertebrates and jawless vertebrates. In contrast, expansion of the pathway members, especially ligands, was observed in jawed vertebrates (gnathostomata) most likely due to 2R event. 3R in teleosts and 4R in common carp were also observed although many TGF-β pathway members were lost after duplication with only a single copy maintained. To be exact, expansion of TGF-β ligands occurred at the early emergence of gnathostomes with retention rates approximately 70%, while it occurred at 3R in teleosts with retention rates of only approximately 20% (Figure 10a). Jawed vertebrates comprise more than 99% of living vertebrate species, including humans. Significant expansion of TGF-β pathway members may set the stage for the generation of key vertebrate evolutionary novelties (like complex brains, heart, blood, bone, cartilage, musculature, and adipose tissue). Three TGFβ isoforms are important for heart development [50,51]. Divergence of *TGFB1*/*2*/*3* in the jawed vertebrates (Figure 10a) has contributed to the formation of genuine heart. Significant expansion of BMPs/GDFs (*BMP2–16*, *GDF1–15*) (Figure 10a) is related not only to bone and cartilage development (such as *BMP2*, *BMP4* and *BMP7*) [52], but also to muscle growth (*GDF8*) [53] and gonadal development and reproduction (*BMP15*, *GDF9*) [42,54]. In addition, a novel subfamily of TGF-β ligands, GDNF, found in the jawed vertebrates (Figure 10a) is crucial for the development of vertebrate nervous system [55]. Additionally, the number of TGF-β ligands greatly exceeded the number of type I and II receptors and smads in jawed vertebrates. This could be best explained by the fact that there are more constraints on intracellular relative to the extracellular components of the signaling pathway [56]. The TGF-β ligands also radiated more than other extracellular protein families (Wnt and Notch families) of parallel importance in developmental specification. The evolutionary success of the TGF-β ligands is in part a result of its large and complex prodomain. This enables complex regulation of biological function during signaling in extracellular environments that can be layered onto cell-surface and intracellular signaling in control of agonism and antagonism [57].

In previous studies, researchers found *BMP16*, a BMP2/4 relative, retained in the genomes of teleosts, was a product of 3R [58]. Subsequently, its presence was reported in a large number of Neopterygii species except in Chondrichthyes and Chondrostei [59]. In the present study, we successfully isolated *BMP16* in whale shark, further supported its 2R origin. Duplication of *TGFB2* (*TGFB2B*), which was considered to be unique to teleosts in previous study [60], was also found in the elephant shark, coelacanth, and python. In line with synergy evolution, the copy of its type II receptor *TGFBR2* (*TGFBR2B*) were not only detected in teleosts but also in elephant shark, coelacanth, spotted gar and tetrapods except mammals. *Acvr1l*, a novel type I receptor first identified in zebrafish [61], was found in the shark, coelacanth, spotted gar and other teleosts as well. Two *SMAD4* genes were found in coelacanth, spotted gar and xenopus, and synteny analyses support their origin from genome duplication. Duplication of *SMAD6* (*SMAD6B*) was also found in coelacanth, xenopus, python, and gecko. These phylogeny and synteny analyses results demonstrated that the duplication of the genes mentioned above all occurred from 2R. Orthologs of most TGF-β genes in vertebrates can be identified in invertebrates even though they experienced several rounds of WGD during evolution. For instance, the origin of the type I receptor can be traced to ALK1/2 and ALK4/5/7 in invertebrates, sequentially ALK4/5/7 underwent duplication forming ALK3/6, then they underwent 2R and 3R event. Only one type II receptor gene (*ACVR2*) was found in the early metazoan sponge and trichoplax indicating that it may be the ancestor-like molecule of type II receptors. Additionally, there were four kinds of smads, including SMAD1/5/8, SMAD2/3, SMAD4 and SMAD6/7 in invertebrates, then a whole SMAD family (SMAD1–8) generated after 2R (Figure 10b). However, some genes arose de novo at different stages of vertebrate evolution, such as *AMH* and subfamily GDNF in gnathostomes and *GSDF* in actinopterygians (Figure 10a).

### 3.2. Roles of TGF-β Pathway Members in Heart, Liver, Especially in Gonads

In the present study, transcriptomic analyses revealed that the majority of TGF-β superfamily members expressed in at least one tissue in tilapia, indicating their essential roles in physiological processes and homeostasis. *tgfb1a*, *tgfb3b*, *tgfbr2*, *bmp4*, *bmp10a*, *bmp10b*, *bmp16* and *smad7* were preferentially expressed in heart as reported in previous studies [58,65,66,67,68,69,70]. We also found *inhbba*, *bmp5* and *bmp6* were prominently expressed in heart. *Gdf8b* was found to be mainly expressed in the muscle. In mice, the physiological role of *Gdf8*, also known as myostatin, was to prevent overgrowth of muscle tissue [71]. *Bmp9*, expressed exclusively in the liver of tilapia, was reported to be required for liver cancer cell growth [72]. Our results also demonstrated that *inhbe* was prominently expressed in liver, which was consistent with the results in mouse and rat [73,74]. Overexpression of *inhbe* in the mouse liver can inhibit regenerative deoxyribonucleic acid synthesis of hepatic cells [75]. It was worth noting that most smads and receptors were expressed in multiple tissues while ligands exhibited a tissue-specific expression. Combining the diversity of ligands, it is possible that different ligands worked for different tissues, while the intracellular components were shared.

In tilapia gonads, *bmp15*, *gdf9*, *gdf3*, *admp2*, *acvr2ba*, *bmp7a*, *smad1*, *smad5* and *smad8* were identified as highly expressed genes in ovary, while *gsdf*, *inha*, *amh* and *amhr2* were identified as highly expressed genes in testis. Generally, there are four key biological events occur during gonadal development of the tilapia: sex determination and differentiation at 5 dah, initiation of germ cell meiosis in ovary at 30 dah, initiation of germ cell meiosis in testis at 90 dah, and vitellogenesis in ovary and sperm maturation in testis at 180 dah [76]. Most XX-enriched genes were expressed in the germ cells of the ovary (*bmp15*, *gdf9*, *gdf3* and *smad1*/*5*/*8*) and their expressions peaked at 180 dah indicating they are important for oogenesis. For instance, *BMP15* and *GDF9* are oocyte-specific growth factors which play important roles in granulosa cell development and fertility in animal models [77]. Deletion of *Gdf9* results in arrest of folliculogenesis at the primary follicle stage and complete infertility in female mice [78] because the cuboidal granulosa cells fail to proliferate [54]. *Admp2* and *acvr2ba* are also expressed highly in the ovary, indicating their possible participation in ovary development. Generally, *SMAD1*/*5*/*8* are regarded as the main responsible smads for downstream mediation of BMP signaling. Existing studies have been focused on the functional role of *SMAD1*/*5*/*8* as a whole, with little research exploring the role of a single smad. Their high expression in ovary from our study implied potential roles in ovary development. Targeted mutation in ovary can help elucidate the roles of these smads. Expressions of the XY-enriched genes started from 5 dah and peaked at 30 dah indicating their possible roles in male sex determination and differentiation. Some have suggested that in vertebrates, only a small number of genes, such as genes belonging to the Dmrt or Sox family, can be recruited to become the master genes of sex determination [38,79,80,81]. However, several novel sex-determining genes coding for TGF-β pathway members were recently identified in teleosts. These included a tandem duplicate of *amh* on the Y chromosome (*amhy*) in tilapia (*Oreochromis niloticus*) [45], a Y-linked duplicate of the *amh* (*amhy*) in pejerrey (*Odontesthes hatcheri*) [44], *gsdf* (*gsdf^Y^*) in medaka (*Oryzias luzonensis*) [46] and *gdf6* (*gdf6^Y^*) in killifish (*Nothobranchius furzeri*) [47]. Even in *Oryzias latipes* and *Oryzias dancena* which use *Dmy* and *Sox3^Y^* as sex-determining gene, respectively, mutation of *gsdf* and *amhr2* also induced sex reversal [43,82]. Overexpressing *gsdf*, a ray-finned fish-specific gene, in medaka and tilapia developing gonads converted XX individuals into functional males [38,39], while knockout of *gsdf* in XY fish of these two species resulted in male to female sex reversal [40,41] suggesting a conserved role of *gsdf* in testicular development. Additionally, in zebrafish whose sex-determining gene has not yet been identified, female deficiency of *bmp15* switched sex and became fertile males [42]. These data highlight the significant role for TGF-β signaling pathway in teleost sex determination. Additionally, transcriptome data in tilapia showed that *inha* was exclusively expressed in the gonads, with higher expression in the testis than ovary. One possible role of *inha* in the ovary might be a mediator between GTH (gonadotropic hormone, including FSH and LH) and *gdf9* to control oogenesis as already proved in zebrafish [83]. However, its role in the testis need further investigations. *AMH* functions primarily through the type II receptor *AMHR2* [84]. Previous reports suggested that AMH/AMHR2 signaling plays an important role in fish sex determination [43,45,85]. Interestingly enough, *AMHR2* was not identified in Chimaeriformes (elephant shark, whale shark) and Cypriniformes, at least in Cyprinidae (zebrafish, common carp, grass carp, bluntnose black bream), even though they had the ligand *AMH*, which indicated that *AMHR2* probably first appeared in Osteichthyes and was lost secondarily in Cyprinidae after 2R. We speculated that another type II receptor, most likely *BMPR2*, might be recruited as *AMH* receptor in these species. It is worth noting that zebrafish has different sex determination mechanism compared to the other teleosts which may be attributed to the absence of *AMHR2*.

In this study, the temporal and spatial expression profiles from transcriptome data were validated by examination of eight TGF-β pathway genes, *bmp15*, *gdf9*, *gdf3*, *smad1*, *smad5*, *smad8*, *gsdf* and *amh* by qPCR, ISH and IHC. All displayed similar ontogeny expression patterns as those from transcriptome data. In addition, *gsdf* and *amh* were identified as XY-enriched genes in this study, which were consistent with the results reported previously in medaka and tilapia [40,45]. The tissue distribution of TGF-βs and Inhibins in tilapia agreed well with that reported in mammals [86,87,88]. Furthermore, the expression profiles of TGF-β family members based on the gonadal transcriptome data from our group [76] and from adult fish downloaded from NCBI [89] were quite similar. All these results provided useful data for further study of the function of this signaling pathway in the animal kingdom, especially in teleosts.

## 4. Materials and Methods

### 4.1. Identification of TGF-β Pathway Members in Representative Animal Species

We examined the genomes of 24 representative animal species (sponges, trichoplax, jellyfish, tapeworm, nematode, leech, oyster, fruit fly, common urchin, vase tunicate, lancelet, lamprey, elephant shark, coelacanth, spotted gar, tilapia, medaka, fugu, zebrafish, common carp, western clawed frog, Burmese python, chicken and human) to identify TGF-β pathway members in each species. The genomic sequences of all species are available at the NCBI and Ensembl database. All members were identified by tblastn (E = 2 × 10^−5^) against genome sequences, using zebrafish and human TGF-β members as the query sequences.

### 4.2. Phylogenetic Analyses and Genomic Distribution

The amino acid sequences of TGF-β pathway members were aligned by Clustal X with default parameters using the multiple alignment software BioEdit (Carlsbad, USA) [90]. Phylogenetic trees were generated by the maximum likelihood (ML) method using the program MEGA 6.0 software (Tempe, USA) [91] with a bootstrap of 1000 replicates to assess the confidence in the phylogeny [92].

Genomic distribution of TGF-β pathway members was performed using UCSC Blat search (Available online: http://genome.ucsc.edu/cgi-bin/hgBlat) [93]. Position and orientation of smad4 and its adjacent genes on chromosome were determined using Genomicus (Available online: http://www.genomicus.biologie.ens.fr/genomicus-89.01/cgi-bin/search.pl) [94].

### 4.3. Expression Analyses of Tilapia TGF-β Pathway Members in Adult Tissues and Gonads at Different Developmental Stages

The transcriptomes of brain, heart, liver, ovary, testis, kidney, muscle and head kidney of adult tilapia were downloaded from the NCBI database (Accession codes: PRJNA78915 and SRR1916191) [4,89]. A normalized measure of FPKM (fragments per kilobase of exon per million fragments mapped) was used to normalize the expression profiles of TGF-β pathway members. TGF-β pathway members with a threshold of RPKM value ≥ 1.25 in each tissue or the total FPKM ≥ 10 in all eight tissues were used to determine a reasonable expression [95]. Bidirectional hierarchical clustering analyses were performed using the pheatmap package (Available online: https://cran.r-project.org/web/packages/pheatmap/index.html) in R language to cluster gene expression values and tissues [96,97]. Tissue distribution (FPKM) of TGF-β pathway members in eight adult tissues of tilapia are listed in Table S3.

Eight pairs of XX and XY gonadal transcriptomes from tilapia at eight developmental stages, 5, 7, 20, 30, 40, 90, 180, 300 dah (days after hatching) were sequenced in our previous study [88]. TGF-β pathway members with RPKM < 1.25 in each sample or the total FPKM < 20 in 16 transcriptomes from eight developmental stages were considered arbitrarily as background expression. Standards and identification of XX/XY-enriched genes of the TGF-β pathway members were performed as described previously [95]. Two-tailed Student’s t-test was used to compare the expression difference of TGF-β pathway members between testis and ovary, and *p* < 0.05 was considered statistically significant.

### 4.4. Validation of Expression Profile of TGF-β Pathway Members by qPCR and Cellular Location by ISH and IHC

Ontogeny expression of *bmp15* and *gdf9* was examined by qPCR. Gonads were dissected from XX and XY tilapia at 5, 30, 90, 180 dah (about 2–200 gonads were collected at each time point depending on the gonad size). Three replicates were uesd for each stage. The mRNAs were extracted and reverse transcribed into cDNA according to the protocol described previously [98]. qPCR examination was carried out according to the manufacturer’s protocol of The SYBR Green I Master Mix (TaKaRa, Dalian, China). Three genes (*β-actin*, *gapdh* and *eef1a1a*) of tilapia were used as internal controls to normalize the expression of the two genes. The Data are presented as mean ± SD. Statistical significance was defined by one-way analysis of variance (ANOVA) followed by post hoc test using the statistical package GraphPad Prism (GraphPad Software, Inc., La Jolla, USA), and *p* < 0.05 was considered statistically significant.

*Bmp15*, *gdf9*, *gdf3* and *smad1*/*5*/*8* were XX-enriched genes from our transcriptome data. To ascertain which population of cells in gonads expressed these genes, ISH was performed using tilapia ovaries and testes at 180 dah. The fixation, embedding, sectioning of dissected gonads and ISH were performed as described previously [99]. Probes of sense and antisense digoxigenin (DIG)-labeled RNA strands were transcribed in vitro from linearized pGEM-T easy-*bmp15*/*gdf9*/*gdf3*/*smad1*/*smad5*/*smad8* cDNA using a RNA labeling kit (Roche, Mannheim, Germany).

*Gsdf* and *amh* were XY-enriched genes from our transcriptome data. To locate the cellular expression pattern of the two genes in the developing gonads, IHC was performed using tilapia ovaries and testes at 5, 30, 90 and 180 dah. Gonads were collected and fixed in Bouin’s solution overnight, and then dehydrated, embedded in paraffin and sectioned at 5 μm thickness. Gsdf and Amh polyclonal antibodies for tilapia prepared by our group were used to examine their respective expression in tilapia gonads at 1:1000 and 1:500 dilution, respectively [41,45]. For the negative control, the primary antibody was replaced with normal rabbit serum. The sections were incubated with a second antibody (goat anti-rabbit IgG) conjugated with horseradish peroxidase (Bio-Rad, Hercules, CA, USA) at 1:2000. Immunoreactive signals were visualized using diaminobenzidine (Sigma, Burlington, MA, USA) as substrate. An Olympus BX51 light microscope (Olympus, Tokyo, Japan) was used to image the stained sections. Sequences of primers and probes used for qPCR and ISH, respectively, are listed in Table S4.

## 5. Conclusions

In summary, we have performed a detailed mining of TGF-β signaling pathway across 24 animal species genomes. As shown in Figure 10, the TGF-β signaling pathway appeared in the earliest-diverged multicellular animals. The total number of TGF-β ligands, receptors and smads changed slightly in invertebrates and jawless vertebrates. In contrast, expansion of the pathway members, especially ligands, was observed in jawed vertebrates due to 2R event. Duplications of TGF-β members were also observed from 3R in teleosts. Type II receptors may be originated from the *ACVR2*-like ancestor. Additionally, transcriptome data showed that most smads and receptors were expressed in multiple tissues while the ligands exhibited a tissue-specific expression especially in heart and gonads. Combining the diversity of ligands, it is possible that the intracellular components were shared by different ligands. Taken together, these results present a thorough overview of TGF-β signaling pathway and provide a new perspective on the origin, evolution, and expression of this family in the animal kingdom, especially in vertebrates.

## Supplementary Materials

Supplementary materials can be found at http://www.mdpi.com/1422-0067/19/4/1154/s1.

## Abbreviations

WGD	whole genome duplication
TGF-β	transforming growth factor β
BMP	bone morphogenetic proteins
GDF	growth and differentiation factor
1R	first round of genome duplication
2R	second round of genome duplication
3R	third round of genome duplication
4R	fourth round of genome duplication
SMAD	composite name from Sma (*Caenorhabditis elegans*) and Mad (*Drosophila melanogaster*)
R-SMAD	receptor-regulated smad
Co-SMAD	common smad
I-SMAD	inhibitory smad
ALK	activating receptor-like kinase
Gsdf	gonadal soma-derived factor
Amh	anti-Müllerian hormone
RNA-seq	RNA sequencing
SRA	Sequence Read Archive
FPKM	fragments per kilobase of exon per million fragments mapped
ISH	in situ hybridization
qPCR	quantitative real time polymerase chain reaction
RT-PCR	reverse transcription PCR
