# Supplementary material for: Identification and Evolution of TGF-β Signaling Pathway Members in Twenty-Four Animal Species and Expression in Tilapia
Source: Int J Mol Sci. 2018 Apr 11;19(4):1154. doi: 10.3390/ijms19041154 (PMC5979292; doi:10.3390/ijms19041154)
Supplement: Supplementary file 1 [file ijms-19-01154-s001.zip › Table S4.docx]

**Table S4. All primer sequences used in present study.**

| **Primer** | **Sequence** | **Purpose** |
| --- | --- | --- |
| *bmp15*-qF | 5′-ACGAGCAGAAAGCGGACCAGA-3′ | qPCR |
| *bmp15*-qR | 5′-GCGGAGAAGAGCGAAGGTGAAC-3′ |  |
| *gdf9*-qF | 5′-CTGTCCGACTGATCAAGCCACAAG-3′ |  |
| *gdf9*-qR | 5′-CGACCGCTCATACTCCTGGATAGT-3′ |  |
| *β-actin*-qF | 5′-GGCATCACACCTTCTACAACGA-3′ | internal control |
| *β-actin*-qR | 5′-ACGCTCTGTCAGGATCTTCA-3′ |  |
| *gapdh-qF* | 5′-AAGCTCATTTCCTGGTAT-3′ |  |
| *gapdh-qR* | 5′-CCTTTGCTGATTTCCTTG-3′ |  |
| *bmp15*-ISH-F | 5′-GCTTAGGCTTCCTGCGTGTCTG-3′ | *in situ* hybridization |
| *bmp15*-ISH-R | 5′-CGTGTGCGGTGATGTCTGTCTC-3′ |  |
| *gdf3*-ISH-F | 5'-GTTGCTGCTGGCGGTGTGTT-3' |  |
| *gdf3*-ISH-R | 5'-ATGGTGACGGAGGTGGATTCGG-3' |  |
| *gdf9*- ISH-F | 5'-GATGTCAGTTGCCCTTCGTCGTT-3' |  |
| *gdf9*- ISH-F | 5'-GACCGCTCATACTCCTGGATAGTGA-3' |  |
| *smad1-* ISH-F | 5'-CCAACACCTTGCCTTTCCCACAT-3' |  |
| *smad1-* ISH-R | 5'-TGGACGAAGATACTGCTGTCACTC-3' |  |
| *smad5-* ISH-F | 5'-TACCACGGCACAGCGAGTTCA-3' |  |
| *smad5-* ISH-R | 5'-GCCTCTCCGACACGATTGTTAAGTT-3' |  |
| *smad8-* ISH-F | 5'-AGTGCTGCCACCAGTCCTTGT-3' |  |
| *smad8-* ISH-R | 5'-TCTGTGAAGCCGTCAACCAGGA-3' |  |
