# Supplementary figures and images for: Identification and Evolution of TGF-β Signaling Pathway Members in Twenty-Four Animal Species and Expression in Tilapia
Source: Int J Mol Sci. 2018 Apr 11;19(4):1154. doi: 10.3390/ijms19041154 (PMC5979292; doi:10.3390/ijms19041154)

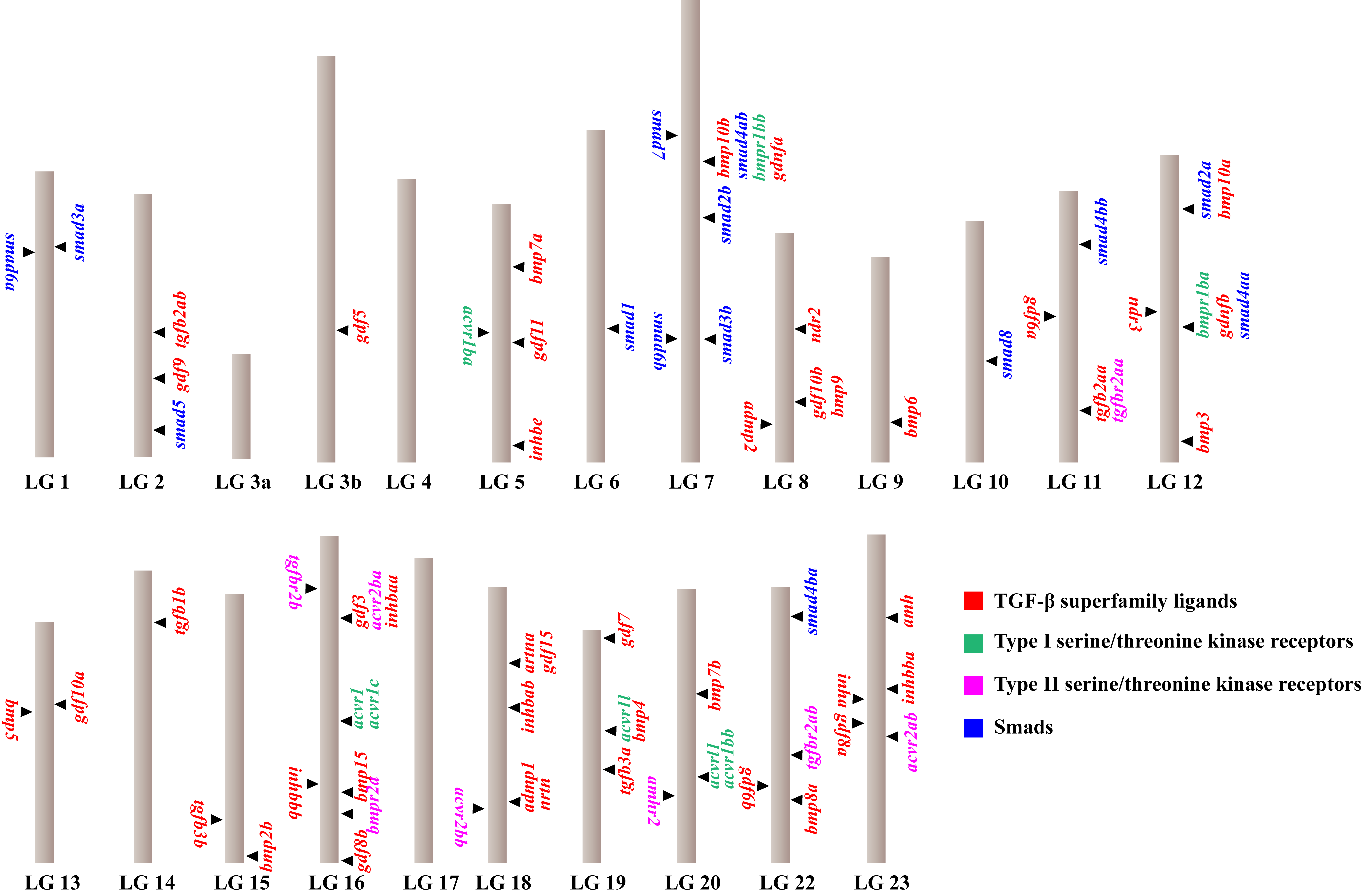

Supplement: Supplementary file 1 [file ijms-19-01154-s001.zip › Figure S1.tif]

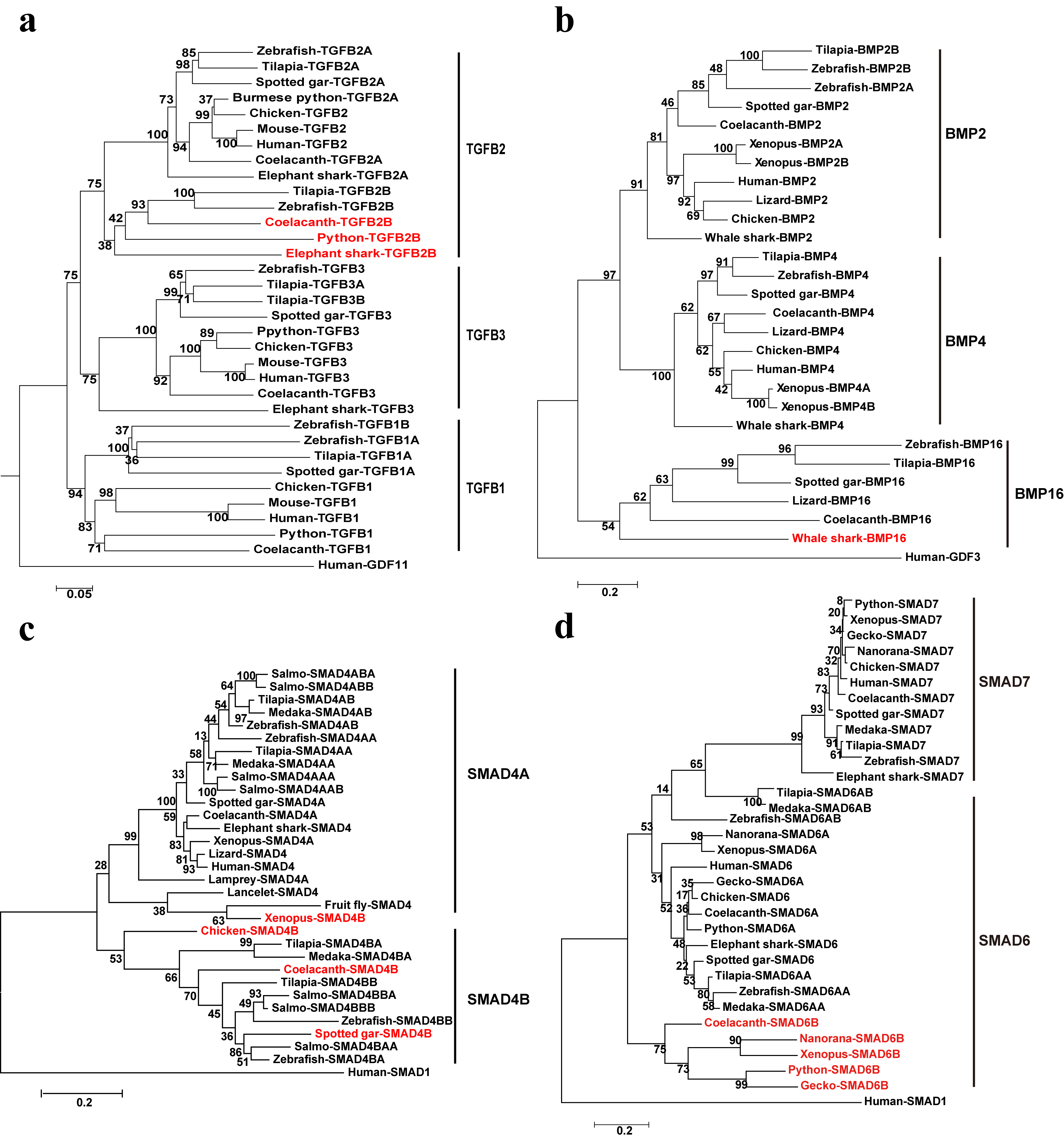

Supplement: Supplementary file 1 [file ijms-19-01154-s001.zip › Figure S2.tif]

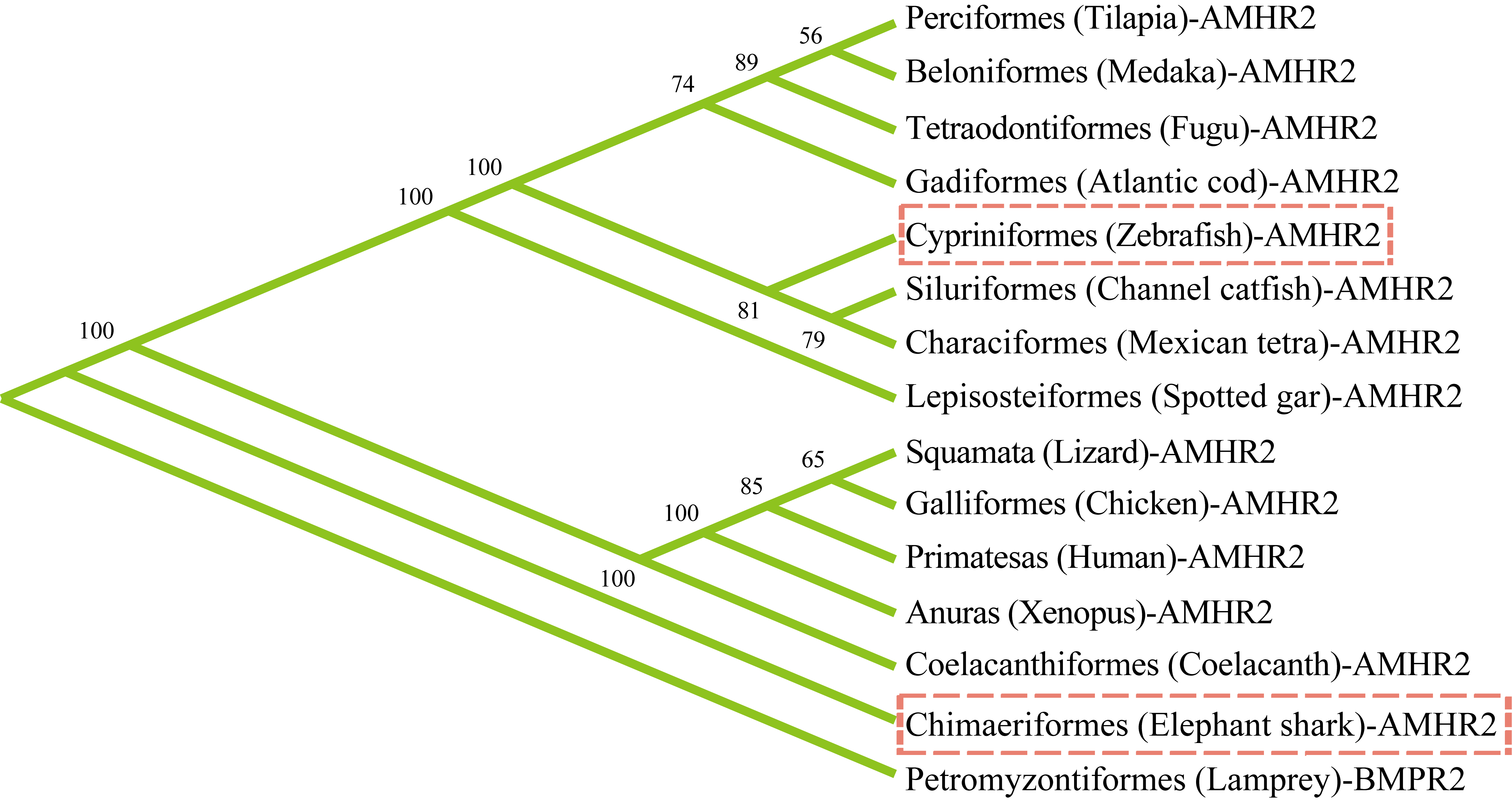

Supplement: Supplementary file 1 [file ijms-19-01154-s001.zip › Figure S3.tif]

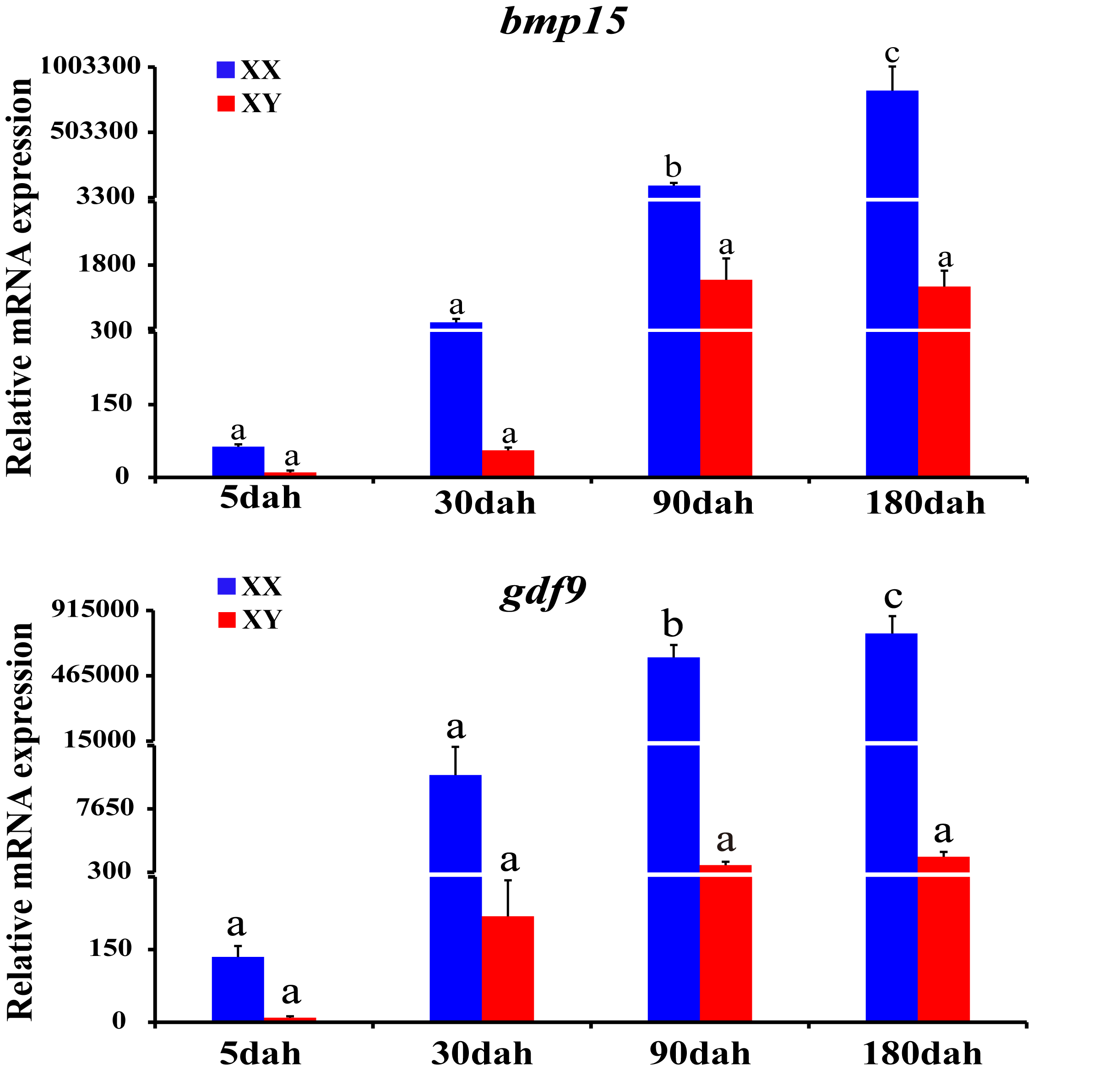

Supplement: Supplementary file 1 [file ijms-19-01154-s001.zip › Figure S4.tif]

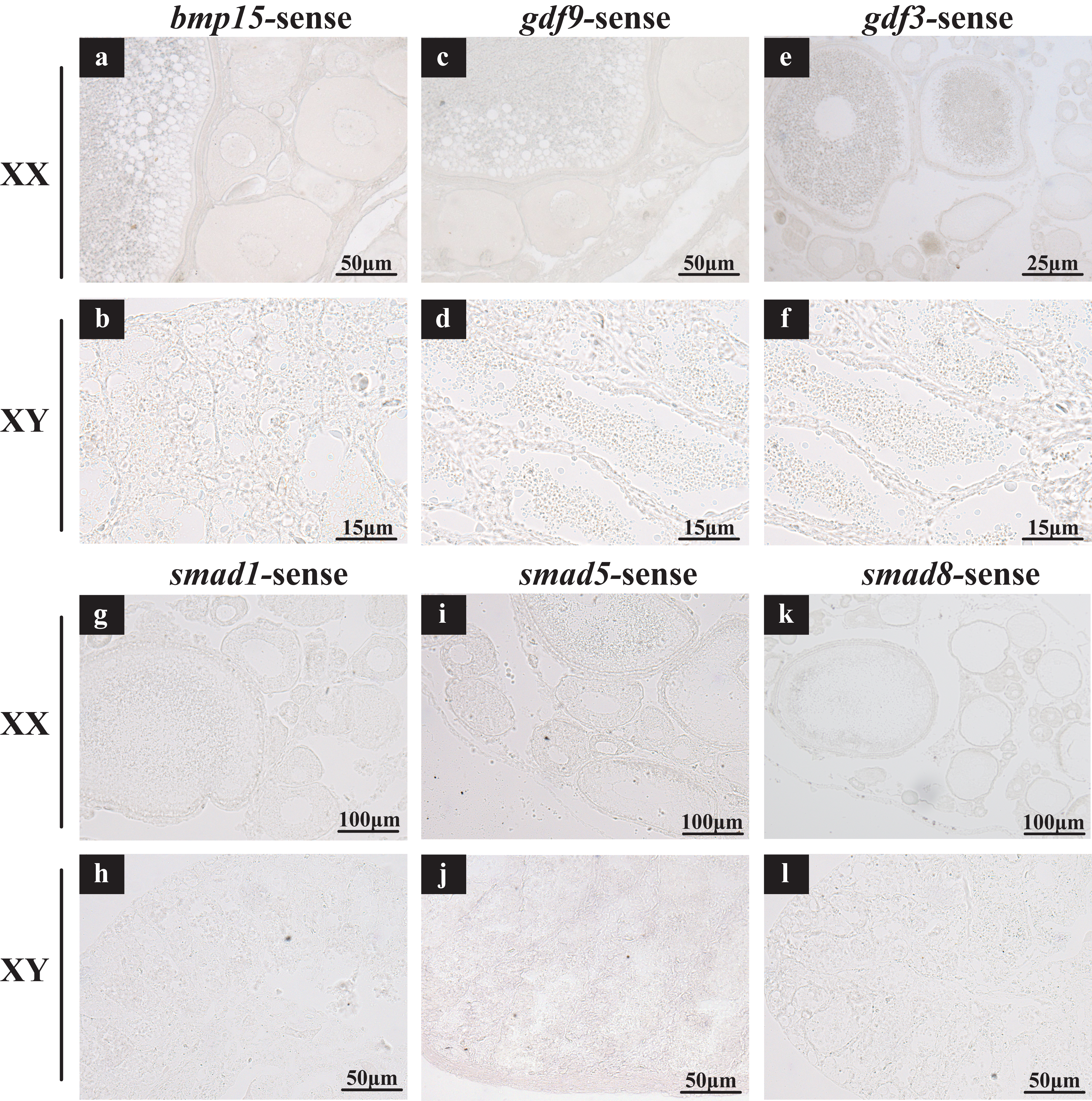

Supplement: Supplementary file 1 [file ijms-19-01154-s001.zip › Figure S5.tif]

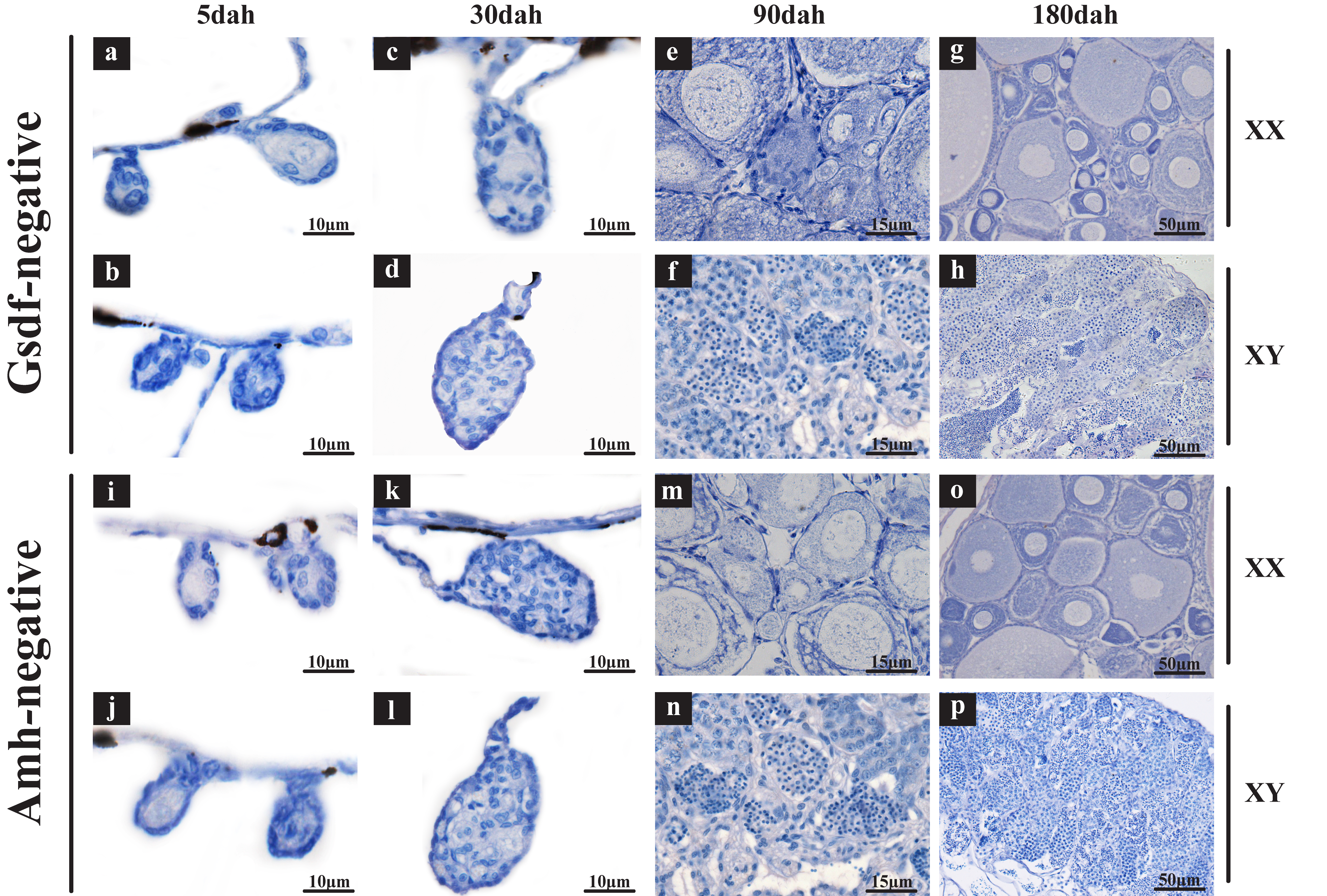

Supplement: Supplementary file 1 [file ijms-19-01154-s001.zip › Figure S6.tif]
