# Supplementary material for: Identification and Evolution of TGF-β Signaling Pathway Members in Twenty-Four Animal Species and Expression in Tilapia
Source: Int J Mol Sci. 2018 Apr 11;19(4):1154. doi: 10.3390/ijms19041154 (PMC5979292; doi:10.3390/ijms19041154)
Supplement: Supplementary file 1 [file ijms-19-01154-s001.zip › Supplemental Figures.doc]

**Supplemental Figures Legends**

**Figure S1. Genomic distribution of TGF-β family members in tilapia.** Results showed that 74 distinct genes were mapped to tilapia different linkage groups (LG) except LG3a, LG4 and LG7. Additionally, the location of the other 6 genes, i.e., *bmp16*, *lft1*, *tgfbr1b*, *bmpr1aa*, *bmpr2b* and *acvr2aa* was unknown.

**Figure S2.** **Phylogenetic tree of TGFB2 (a), BMP16 (b), SMAD4 (c) and SMAD6 (d).** The ML method was used to construct the tree by MEGA 6.0 software. The multiple alignment software Bioedit was used to align the amino acid sequences. (a) BMP16 was identified for the first time in whale shark in this study. (b) Duplication of TGFB2 was found in the elephant shark, coelacanth and python. (c) Phylogeny analysis of SMAD4 indicated that it has undergone 2R event to form two copies in coelacanth, spotted gar and xenopus. After 3R event, most teleosts had 4 SMAD4 copies. (d) SMAD6B was found in coelacanth, xenopus, python and gecko.

**Figure S3. Phylogenetic tree of AMHR2.** The tree was constructed with the amino acid sequence of AMHR2 using the same method as described in figure S2. Lamprey BMPR2 was used as an outgroup. AMHR2 was not identified in Chimaeriformes (elephant shark, whale shark) and Cypriniformes, at least in Cyprinidae (zebrafish, common carp, grass carp and bluntnose black bream) genomes which were denoted with the dotted box.

**Figure S4. Ontogeny expression of *bmp15* and *gdf9* in tilapia by qPCR (n=3)**. Data were expressed as the mean±SD. Different letters indicated statistical differences at P<0.05 as determined by one-way ANOVA followed by post hoc test.

**Figure S5. Negative controls for ISH staining of gonadal sections from tilapia at 180 dah using sense probes of 6 TGF-β pathway genes.** No positive signals were observed in all sections hybridized with sense probes. Scale bar: a, c, h, j and l 50 µm; b, d and f 15 µm; e 25 µm, g, i and k 100 µm.

**Figure S6.** **Negative controls for IHC staining of Amh and Gsdf in tilapia gonads at different stages.** No signals were observed in all sections with normal rabbit serum as primary antibody. Scale bar: a-d and i-l 10 µm; e, f, m and n 15 µm; g, h, o and p 50 µm.
